# A New Series of Carlactonoic Acid Based Strigolactone Analogs for Fundamental and Applied Research

**DOI:** 10.3389/fpls.2020.00434

**Published:** 2020-04-15

**Authors:** Muhammad Jamil, Boubacar A. Kountche, Jian You Wang, Imran Haider, Kun-Peng Jia, Ikuo Takahashi, Tsuyoshi Ota, Tadao Asami, Salim Al-Babili

**Affiliations:** ^1^The BioActives Lab, Center for Desert Agriculture, Biological and Environment Science and Engineering (BESE), King Abdullah University of Science and Technology, Thuwal, Saudi Arabia; ^2^Graduate School of Agricultural and Life Sciences, The University of Tokyo, Tokyo, Japan

**Keywords:** strigolactones, *Striga*, *Phelipanche*, tillering, senescence, strigolactone analogs

## Abstract

Strigolactones (SLs) are a group of carotenoid derived plant hormones that play a key role in establishing plant architecture and adapting it to environmental changes, and are involved in plants response to biotic and abiotic stress. SLs are also released into the soil to serve as a chemical signal attracting beneficial mycorrhizal fungi. However, this signal also induces seed germination in root parasitic weeds that represent a major global threat for agriculture. This wide spectrum of biological functions has made SL research one of the most important current topics in fundamental and applied plant science. The availability of SLs is crucial for investigating SL biology as well as for agricultural application. However, natural SLs are produced in very low amounts, and their organic synthesis is quite difficult, which creates a need for efficient and easy-to-synthesize analogs and mimics. Recently, we have generated a set of SL analogs, Methyl Phenlactonoates (MPs), which resemble the non-canonical SL carlactonoic acid. In this paper, we describe the development and characterization of a new series of easy-to-synthesize MPs. The new analogs were assessed with respect to regulation of shoot branching, impact on leaf senescence, and induction of seed germination in different root parasitic plants species. Some of the new analogs showed higher efficiency in inhibiting shoot branching as well as in triggering parasitic seed germination, compared to the commonly used GR24. MP16 was the most outstanding analog showing high activity in different SL biological functions. In summary, our new analogs series contains very promising candidates for different applications, which include the usage in studies for understanding different aspects of SL biology as well as large scale field application for combating root parasitic weeds, such as *Striga hermonthica* that devastates cereal yields in sub-Saharan Africa.

## Introduction

Strigolactones (SLs) are well-known carotenoid derived metabolites that act as endogenous phyto-hormones as well as rhizospheric signaling molecules. As plant hormones, SLs are involved in controlling shoot branching/tillering, root architecture and contribute to further aspects of plant growth, as well as to pathogen defense and abiotic stress responses (Gomez-Roldan et al., 2008; Umehara et al., 2008; Agusti et al., 2011; Kapulnik et al., 2011; Ruyter-Spira et al., 2011; Ha et al., 2014; Decker et al., 2017). Upon release into the rhizosphere, SLs stimulate the metabolism of arbuscular mycorrhiza fungi (AMF) and induce germination of their spores and branching of their hyphae (Bonfante and Genre, 2010; Gutjahr and Parniske, 2013; Fiorilli et al., 2019). These changes pave the way for establishing the beneficial AM symbiosis, in which AMF help host plants to uptake mineral nutrients and water through a wide net of extraradical fungal hyphae, and obtain in return sugars and other reduced carbon compounds (Akiyama et al., 2005; Bonfante and Genre, 2010). However, released SLs are also sensed by seeds of root parasitic plants, which use SLs as germination stimulant. These weeds cause enormous losses in yields of several crop species (Cook et al., 1966). In particular, root parasitic plants of the genus *Striga* are considered as one of the major biotic constraints and threats to global food security, devastating cereal production in Africa. It has been reported that severe infestation of *Striga* results 50% to complete crop failure, affecting the life of 300 million people and causing 7 billion US $ loss annually (Gressel et al., 2004; Ejeta, 2007). *Striga* infestation is spreading over 50 million ha of land in 32 African countries (Rodenburg et al., 2016). The dependency of *Striga* seed germination on SL signaling can be exploited to combat this weed by artificial application of SL analogs in the absence of a host, a strategy termed as “suicidal gemination” (Kountche et al., 2019). In more details, *Striga* seeds can be germinated by the application of synthetic SL analogs in infested fields before sowing the crop seeds. The arising *Striga* seedlings would then die after few days of germination due to lack of host needed for the survival of this obligate parasite. In this way, the seed bank of infested soil can be depleted (Kgosi et al., 2012; Zwanenburg et al., 2016a; Kountche et al., 2019).

The first steps in SL biosynthesis take place in plastids and are catalyzed by the 9-*cis*/all-*trans*-β-carotene isomerase (D27) (Alder et al., 2012; Bruno and Al-Babili, 2016; Abuauf et al., 2018) and the two carotenoid cleavage dioxygenases 7 (CCD7) (Alder et al., 2012; Bruno et al., 2014) and CCD8 (Alder et al., 2012; Bruno et al., 2017). Sequential action of these three enzymes leads to the key intermediate carlactone (CL) (Al-Babili and Bouwmeester, 2015; Jia et al., 2018). CL is then released into the cytosol where it is converted by cytochrome P450 (711 clade; homologs of the Arabidopsis MAX1 homologs) and other largely unknown enzymes into canonical and non-canonical SLs (Abe et al., 2014; Seto et al., 2014; Zhang et al., 2014). The conversion of carlactone into SLs proceeds via its oxidation into carlactonoic acid (Alder et al., 2012; Abe et al., 2014; Zhang et al., 2014; Al-Babili and Bouwmeester, 2015; Bruno and Al-Babili, 2016; Bruno et al., 2017; Abuauf et al., 2018). Canonical and non-canonical SLs differ in the structure of the moiety that is coupled to the characteristic SL D-ring in a defined stereo-configuration (Al-Babili and Bouwmeester, 2015; Jia et al., 2018). This moiety consists of a tricyclic lactone (ABC-ring), in case of canonical SLs, and of different less defined structures in the non-canonical ones (Jia et al., 2018). SL signal transduction is mediated by the receptor D14, an α/β-hydrolase that binds and hydrolyzes the SL ligand into the characteristic D-ring and the corresponding second moiety, and an F-box protein (MAX2 in Arabidopsis) (Scaffidi et al., 2014; Waters et al., 2017; Jia et al., 2019).

The isolation and organic synthesis of natural SLs in large quantities is very difficult because of their complex structure and their scarce amount in plant tissues and root exudates (Zwanenburg et al., 2016b, c). Therefore, SL fundamental research, as well as agricultural application, has been heavily relying on synthetic SL analogs. In 1981, the common canonical SL analog GR24 with ABC-ring coupled to the D-ring was developed and since then, has been widely used in labs for SL and root parasitic weed research (Johnson et al., 1981; Mangnus and Zwanenburg, 1992). However, large scale synthesis of GR24 is very expensive and laborious because of its many synthesis steps (Mangnus et al., 1992a, b). To overcome this issue, scientists first explored structure-activity relationship of SLs (Zwanenburg and Pospisil, 2013). These studies showed that the α/β unsaturated system and D-ring of SL analogs are indispensable for their activity, while modifications of the ABC-ring impacted the level of their efficiency, as shown by testing the activity of the structurally less complex analogs GR5 and GR7 (Mangnus et al., 1992b; Zwanenburg et al., 2009, 2016c). Other simple analogs were developed by isosteric replacement leading to imino SL analogs (Kondo et al., 2007), CISA-1 (Rasmussen et al., 2013) and strigolactams (Lachia et al., 2015). We recently reported the synthesis of Nitro-Phenlactones (Jia et al., 2016) and a series of Methyl-Phenlactonoates (MPs) (Jamil et al., 2018, 2019), which were designed based on the structure of carlactone and carlactonoic acid, respectively. In this paper, we further proposed a new series of carlactonoic acid-based SL analogs. In the following, we describe the synthesis and evaluation of this new set of MPs, which is characterized by simple synthesis and includes quite highly efficient SL analogs.

## Materials and Methods

### Plant Materials and Growth Conditions

*Striga hermonthica* seeds were provided by Prof. Abdel Gabar Babiker collected from Sorghum infested field near Wad Medani, Sudan. Seeds of *Phelipanche aegyptiaca* were provided by Prof. Mohamed Ewis Abdelaziz, Cairo University, Egypt. Seeds of the highly *Striga* susceptible rice *cv* IAC 165 are a gift from Dr. Jonne Rodenburg, Africa Rice, Tanzania. Dr. Junko Kyozuka, Tohoku University, Japan provided us seeds of the rice *d10-1* and *d3-1* mutants in the Shiokari background (Ishikawa et al., 2005; Arite et al., 2007, 2009; Hu et al., 2010). Rice seeds were germinated at 30°C on moist filter paper, and seedlings were grown at 30°C and 70% relative humidity with fluorescent white light (130–180 μM m^–2^ s^–1^) 12 h day/night period. *Striga* and *Phelipanche* seeds pre-conditioning was done under moist conditions in the dark at 30 and 22°C, respectively.

### General Procedure for the Preparation of Esters and Amide

**FIGURE 1:**
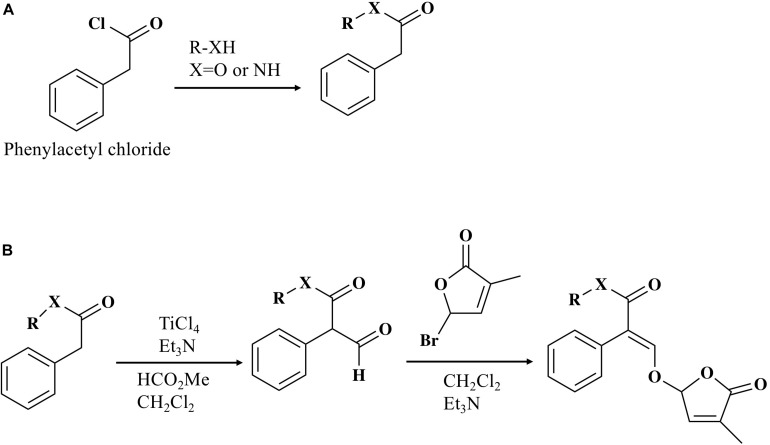
Synthesis of MP16-MP25. **(A)** General procedure for the preparation of esters and amide. **(B)** General procedure for the preparation of MPs (MP16-MP25).

Phenylacetyl chloride (3 mmol) was added dropwise to the solution of pyridine (10 ml) and alcohol or phenol (10 mmol) with stirring in a 50 ml of round-bottom flask in an ice bath (Figure 1). The mixture was stirred at room temperature overnight then poured into ice-cold water (10 ml). The organic layer was extracted with ethyl acetate (5 ml × 3), washed with saturated brine (20 ml), dried over anhydrous sodium sulfate, and filtered. The organic solvent was evaporated under reduced pressure and the residue was then purified on a silica gel (Wakosil^®^C-300HG)column eluted with ethyl acetate/n-hexane to give an ester (53–95% yield). The preparation of amide compound followed the same step as described above, except dimethyl amine was used as the starting material instead of alcohol (78% yield).

### General Procedure for the Preparation of MPs (MP16–MP25)

**FIGURE 2:**
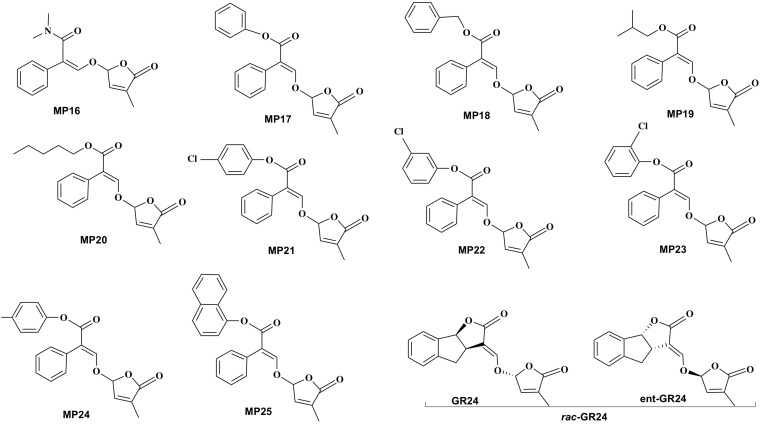
Structure of the developed methyl phenlactonoates (MP16-MP25) and the standard SL analog *rac*-GR24.

To an ice-cold solution of the ester (2.47 mmol) obtained above, methyl formate (7.91 mmol), and trimethylamine (5.93 mmol) in dichloromethane (15 ml), titanium (IV) chloride (4.94 mmol) were added slowly and stirred for 10 min (Figure 1). The mixture was stirred further for 2 h at room temperature and then poured into ice-cold water (10 ml). The organic layer was extracted with ethyl acetate (5 ml × 3), washed with saturated brine (20 ml), dried over anhydrous sodium sulfate, and filtered. The organic solvent was evaporated under reduced pressure and the residue was then purified on a silica gel (Wakosil^®^C-300HG) column eluted with ethyl acetate/n-hexane to give aldehyde. All of the compounds thus obtained were used in the next step without measuring weight. To an ice-cold solution of the aldehyde obtained above in dichloromethane (13 ml), 5-bromo-3-methyl-2(5H)-furanone (2.47 mmol) and trimethylamine (3.71 mmol) was added and stirred overnight at room temperature and then poured into ice-cold water (10 ml). The organic layer was extracted with ethyl acetate (5 ml × 3), washed with saturated brine (20 ml), dried over anhydrous sodium sulfate, and filtered. The organic solvent was evaporated under reduced pressure and the residue was then purified on a silica gel (Wakosil^®^C-300HG) column eluted with ethyl acetate/n-hexane to give enol ether. MP17-MP25 (Figure 2) were prepared according to the method above (10–67% yield). The preparation of MP16 followed the same step as described above, except the amide compound was used as the starting material instead of ester (21% yield). ^1^H, ^13^C NMR and HRMS spectra of all synthesized compounds were recorded on JEOL JNM-ECA500II 500 MHz spectrometer and ABSciex TripleTOF5600 Q-TOF LC/MSMS. *Z/E* stereochemistry was assigned based on NOE experiments. In case of the E-isomer (MP16) NOE between vinyl proton and aromatic proton was observed. In case of Z-isomer NOE between vinyl proton and aromatic proton was not observed and instead NOE between vinyl proton and methyl proton of carbomethoxy group was observed.

The physico-chemical properties of all MPs are as under:

**MP16** (*Z*)-N,N-Dimethyl-3-((4-methyl-5-oxo-2,5-dihydrofuran-2-yl)oxy)-2-phenylacrylamide.

^1^H NMR (500MHz, CDCl_3_): δ 7.27(5H, m), 6.93(1H, s), 6.77(1H, s), 6.09(1H, s), 3.03(3H, s), 2.91(3H, s), 1.97(3H, s). ^13^C-NMR (126 MHz, CDCl3) δ: 170.86, 167.15, 142.09, 139.30, 134.76, 132.93, 128.78, 127.58, 125.27, 121.11, 100.25, 37.54, 34.27, 10.51. HRMS (m/z): [M+H]^+^ calcd. for C_16_H_18_NO_4_, 288.1230; found, 288.1231. 21% Yield.

**MP17** (*E*)-Phenyl 3-((4-methyl-5-oxo-2,5-dihydrofuran-2-yl)oxy)-2-phenylacrylate.

^1^H NMR (500MHz, CDCl_3_): δ 7.98(1H, s), 7.40(5H, m), 7.32(1H, m), 7.24(1H, t, *J* = 7.5Hz), 7.38(2H, d, *J* = 5.5Hz), 6.90(1H, s), 6.20(1H, s), 2.00(3H, s). ^13^C-NMR (126 MHz, CDCl3) δ: 170.32, 165.27, 154.13, 150.62, 141.17, 135.26, 131.14, 130.03, 129.25, 127.81, 127.67, 125.64, 121.59, 114.93, 100.52, 10.49. HRMS (m/z): [M+H]^+^ calcd. for C_20_H_17_O_5_, 337.1071; found, 337.1069. 38% Yield.

**MP18** (*E*)-Benzyl 3-((4-methyl-5-oxo-2,5-dihydrofuran-2-yl)oxy)-2-phenylacrylate.

^1^H NMR (500MHz, CDCl_3_): δ 7.76(1H, s), 7.33(10H, m), 6.84(1H, s), 6.12(1H, s), 5.22(2H, s), 1.96(3H, s). HRMS (m/z): [M+Na]^+^ calcd. for C_21_H_18_NaO_5_, 373.1046; found, 373.1051. 67% Yield.

**MP19** (*E*)-Isobutyl 3-((4-methyl-5-oxo-2,5-dihydrofuran-2-yl)oxy)-2-phenylacrylate.

^1^H NMR (500MHz, CDCl_3_): δ 7.73(1H, s), 7.32(5H, m), 6.86(1H, s), 6.14(1H, s), 3.96(2H, d, *J* = 5.5Hz), 1.97(3H, s), 1.95(1H, m), 0.91(6H, d, *J* = 7.0Hz). ^13^C-NMR (126 MHz, CDCl3) δ: 170.34, 166.41, 152.93, 141.21, 135.89, 135.07, 131.41, 129.94, 128.36, 127.98, 127.89, 127.67, 127.44, 115.34, 100.40, 66.26, 10.38. HRMS (m/z): [M+H]^+^ calcd. for C_18_H_21_O_5_, 317.1384; found, 317.1381. 28% Yield.

**MP20** (*E*)-Pentyl 3-((4-methyl-5-oxo-2,5-dihydrofuran-2-yl)oxy)-2-phenylacrylate.

^1^H NMR (500MHz, CDCl_3_): δ 7.73(1H, s), 7.33(5H, m), 6.87(1H, s), 6.15(1H, s), 4.17(2H, t, *J* = 7.0Hz), 1.98(3H, s), 1.66(2H, m), 1.33(4H, m), 0.91(3H, t, *J* = 7.5Hz). ^13^C-NMR (126 MHz, CDCl3) δ: 170.34, 166.58, 152.36, 141.31, 1334.95, 131.52, 129.86, 127.52, 127.25, 115.52, 100.35, 64.68, 28.05, 27.86, 22.03, 13.72, 10.32. HRMS (m/z): [M+H]^+^ calcd. for C_19_H_23_O_5_, 331.1540; found, 331.1539. 42% Yield.

**MP21** (*E*)-4-Chlorophenyl 3-((4-methyl-5-oxo-2,5-dihydrofuran-2-yl)oxy)-2-phenylacrylate.

^1^H NMR (500MHz, CDCl_3_): δ 7.96(1H, s), 7.30–7.40(7H), 7.07(2H, d, *J* = 8.5Hz), 6.90(1H, s), 6.20(1H, s), 1.99(3H, s). ^13^C-NMR (126 MHz, CDCl3) δ: 170.23, 165.01, 154.44, 149.06, 141.07, 135.27, 130.91, 130.88, 129.96, 129.23, 127.83, 127.73, 122.99, 114.63, 100.49, 10.45. HRMS (m/z): [M+H]^+^ calcd. for C_20_H_16_ClO_5_, 371.0681; found, 371.0675. 11% Yield.

**MP22** (*E*)-3-Chlorophenyl 3-((4-methyl-5-oxo-2,5-dihydrofuran-2-yl)oxy)-2-phenylacrylate.

^1^H NMR (500MHz, CDCl_3_): δ 7.96(1H, s), 7.39(4H, d, *J* = 4.5Hz), 7.33(2H, m), 7.22(1H, d, *J* = 8.0Hz), 7.18(1H, t, *J* = 2.5Hz), 7.05(1H, d, *J* = 8.0Hz), 6.90(1H, s),6.21(1H, s), 2.00(1H, s). ^13^C-NMR (126 MHz, CDCl3) δ: 170.17, 164.75, 154.61, 150.97, 141.08, 135.03, 134.21, 130.81, 129.93, 129.87, 127.73, 127.62, 125.72, 122.12, 119.98, 114.31, 100.48, 10.29. HRMS (m/z): [M+Na]^+^ calcd. for C_20_H_15_ClNaO_5_, 393.0500; found, 393.0498. 10% Yield.

**MP23** (E)-2-Chlorophenyl 3-((4-methyl-5-oxo-2,5-dihydrofuran-2-yl)oxy)-2-phenylacrylate.

^1^H NMR (500MHz, CDCl_3_): δ 8.03(1H, s), 7.45(3H, d, *J* = 8.5Hz), 7.38(2H, t, *J* = 7.0Hz), 7.25–7.34(2H), 7.19(2H, m), 6.90(1H, s), 6.21(1H, s), 2.00(3H, s). ^13^C-NMR (126 MHz, CDCl3) δ: 170.35, 164.40, 154.56, 146.95, 141.12, 135.53, 130.92, 130.17, 130.15, 127.91, 127.83, 127.68, 126.92, 126.90, 123.89, 114.51, 100.57, 10.62. HRMS (m/z): [M+Na]^+^ calcd. for C_20_H_15_ClNaO_5_, 393.0500; found, 393.0501. 32% Yield.

**MP24** (*E*)-p-Tolyl 3-((4-methyl-5-oxo-2,5-dihydrofuran-2-yl)oxy)-2-phenylacrylate.

^1^H NMR (500MHz, CDCl_3_): δ 7.95(1H, s), 7.36(5H, m), 7.17(2H, d, *J* = 8.0Hz), 6.89(1H, s), 6.20(1H, s), 2.34(3H, s), 1.99(3H, s). ^13^C-NMR (126 MHz, CDCl3) δ: 170.51, 165.65, 153.99, 148.54, 141.26, 135.71, 135.48, 131.34, 130.26, 129.98, 128.02, 127.87, 121.44, 115.42, 100.67, 20.97, 10.81. HRMS (m/z): [M+H]^+^ calcd. for C_21_H_19_O_5_, 351.1227; found, 351.1223. 10% Yield.

**MP25** (*E*)-naphthalen-1-yl 3-((4-methyl-5-oxo-2,5-dihydrofuran-2-yl)oxy)-2-phenylacrylate.

^1^H NMR (500MHz, CDCl_3_): δ 8.11(1H, s), 7.84(2H, m), 7.74(1H, d, *J* = 8.0Hz), 7.28–7.54(9H), 6.92(1H, s), 6.24(1H, s), 2.00(3H, s). ^13^C-NMR (126 MHz, CDCl3) δ: 170.38, 165.38, 154.33, 146.64, 141.10, 135.57, 134.58, 131.21, 130.16, 128.00, 127.98, 127.87, 126.91, 126.46, 126.39, 125.93, 125.37, 121.21, 118.14, 115.14, 100.62, 10.66. HRMS (m/z): [M+H]^+^ calcd. for C_24_H_19_O_5_, 387.1227; found, 387.1209. 55% Yield.

### Rice Micro Tillering Bioassays

After sterilization with 50% sodium hypochlorite, rice seeds (*d10-1*/*ccd8* and *d3-1* mutants) were germinated on moist filter paper in the dark at 30°C. The germinated seeds were then transferred to light in a growth cabinet with fluorescent white light (130–180 μM m^–2^ s^–1^) at 30°C for 7 days. One-week old rice seedlings were shifted to 50 ml falcon tubes (one seedling per tube) filled with half-strength modified Hoagland nutrient solution. The tubes with rice seedlings were kept in green house to grow at 30°C and 70% humidity. After 1 week, the rice seedlings were treated with 2.5 μM of each SL analog. Mock and GR24 (2.5 μM) were included as control treatments. Each of the MPs was applied twice per week up to 3 weeks. Number of tillers per plant, plant height and dry biomass were measured after 3 weeks of MPs application at final harvest.

### Dark-Induced Rice Leaf Senescence

Rice seeds (cv IAC-165) were surface sterilized with 50% sodium hypochlorite solution and 0.05% Tween-20, and germinated on moist filter paper in the sealed petri plates. The petri plates with germinated seeds were transferred to white fluorescent light (130–180 μM m^–2^ s^–1^) with 16 h:8 h (L/D) at 28°C, to establish seedlings for 1 week. Seven days old uniform seedlings were selected and transferred to 50 ml tubes containing half strength modified Hoaglands nutrient solution. After 1 week, 2 cm leaf segments were cut from middle part of third leaves of rice plants. Each segment was put in a well (in 12-well plates) containing 4 ml of 3.0 mM MES buffer with 0.05% Tween-20. MP16 and GR24 were applied at 3.0 μM concentration. Plates were incubated at 30°C in the dark for 7 days. After application of MP16 and GR24, plates were monitored on a daily basis and changes in leaf color, chlorophyll content and ion leakage were monitored.

### Parasitic Seed Germination Bioassays

Seeds of the two root parasitic species *S. hermonthica* and *P. aegyptiaca* were tested for germination in response to MPs applications. After pre-conditioning, as previously described (Jamil et al., 2012), *Striga* and *Phelipanche* seeds were first tested with each MP solutions at 2.5 μM concentration (55 μl per disk) and then at a concentration range from 10^–5^ M to 10^–12^ M, to calculate EC_50_. Sterile MilliQ water and GR24 were applied as a negative and a positive control, respectively. Treated seeds were incubated in dark for 24 h at 30°C (for *S. hermonthica*) and for 1 week at 25°C (for *P. aegyptiaca*). Germination was recorded under a binocular microscope and used to determine the germination rate (%).

### Stability Measurement

Three selected MPs (MP16, MP18, MP21) and *rac*-GR24 were tested for their chemical stability at 21 ± 1°C in aqueous solution with a pH of 5.5–6.0, as described previously (Jamil et al., 2018). Compound solution (1 mg ml^–1^) was prepared with 175 μl ethanol and 750 μl Mili-Q water. Thereafter, 25 μl Indanol (1 mg ml^–1^, internal standard) was spiked in 975 μl previous prepared solution. The time course of degradation was monitored in about 50 ml aliquots by UPLC analysis using an Agilent HPLC ZORBAX Eclipse XDB-C18 column (3.5 μm, 4.6 × 150 mm) eluted first by 5% acetonitrile in water for 0.5 min then by a gradient from 5 to 100% acetonitrile within 18 min in water, and finally by 100% acetonitrile for 5 min. The column was operated at 40°C at 0.35 ml min^–1^ flow rate. Compounds eluted from the column were detected with a photodiode array detector, and the relative quantity of non-degraded amount was calculated using Indanol as internal standard. Stability was monitored at 24 h intervals up to 3 weeks.

### *In vitro* YLG (Yoshimulactone Green) Assays

Purification of ShHTL7/OsD14 was carried out by adopting the procedure explained previously (Jamil et al., 2018). *In vitro* YLG hydrolysis assays were conducted as described before (Tsuchiya et al., 2015). About 3.0 μM of purified ShHTL7/OsD14 protein was used in a reaction buffer (1X PBS buffer, pH 7.3) with 0.1% dimethyl sulfoxide (DMSO) at a 100 μl volume on a 96-well black plate (Greiner). SL analogs (at range between 0.01 and 50 μM) were co-incubated with 1.0 μM of YLG (Tokyo Chemical Industry Co. Ltd., product number E1238) for 60 min at room temperature. In this competition assay, fluorescent intensity was measured by spectraMax i5 (Molecular Devices) at excitation at 480 nm and detection at 520 nm. The variation in fluorescence recorded over the course of 1 h of YLG incubation in protein-free buffer was subtracted from the data collected in presence of protein. IC_50_values were calculated by using Quest Graph^TM^ IC_50_ Calculator.

### Statistical Analyses

Standard procedure was adopted to collect data for each trait, which were analyzed statistically using statistical software package R (version 3.2.2). One-way analysis of variance (ANOVA) and LSD (Least significant difference) multiple range test were applied to investigate the effect of various MPs for various parameters. Half Maximum Effective Concentration (EC_50_) was calculated using IC_50_ toolkit.^1^

## Results

### Synthesis and Structure of MPs

Synthesis scheme and structure of the developed 10 analogs (MP16 to MP25) is shown in Figures 1, 2, respectively. Figure 2 also depicts the structure of *rac*-GR24 used in this study. Physico-chemical properties of these compounds are described in the section General Procedure for the Preparation of MPs.

### Effect of the New Analogs on Tillering of the Rice *d10*/*ccd8* and *d3* Mutants

**FIGURE 3:**
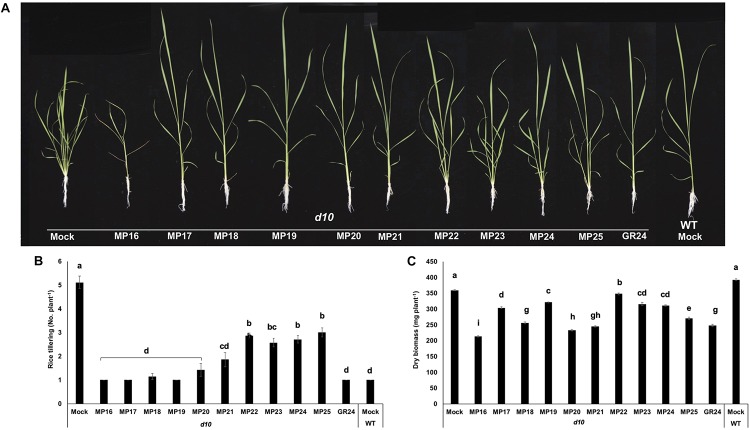
Tillering inhibition of SL deficient *d10*/CCD8 rice mutant by MP16-MP25. **(A)** Tillering phenotype of *d10* mutant in response to MPs. SL analogs were applied (2.5 μM) to 1-week old *d10* rice seedlings grown hydroponically in 50 ml tube twice a week up to 3 weeks. MP16 treatment showed growth retardation and senescence. **(B)** Number of tillers per plant counted after 3 weeks of MPs application. **(C)** Dry biomass of *d10* rice seedlings, measured after 3 weeks of MPs application. Data are means ± SE (*n* = 8). Means not sharing a letter in common differ significantly at *P*_0.05_.

To test their capability in regulating shoot branching, we applied the new analogs at a 2.5 μM concentration to hydroponically grown seedlings of the rice wild type (cv. Shiokari) and its mutants *d10-1*, a SL deficient, high-tillering dwarf mutant disrupted in *CCD8* gene (Arite et al., 2007, 2009), and *d3-1*, a SL insensitive high-tillering dwarf mutant disrupted in *D3* gene that mediates SL signal transduction (Ishikawa et al., 2005; Hu et al., 2010). All MPs showed statistically significant effect in inhibiting tillering of the SL deficient *d10* mutant, compared to mock treatment (Figures 3A–C). Like the standard SL analog GR24, six MPs (MP16-MP21) restored wild-type tillering to the *d10* mutant, decreasing the number of its tillers from 5 (untreated) to 1. As expected, neither MPs nor GR24 showed an effect on the tillering of the SL insensitive *d3* mutant that retained an average of 4 tillers per plant regardless treatment (Supplementary Figure S1). Besides reducing the number of tillers, we observed that MP16 (applied at a 2.5 μM concentration) caused stunted growth and triggered senescence in rice seedlings, leading to reduced biomass and plant height (Supplementary Figure S2). This result indicated that MP16 might possess high activity in growth regulation and senescence.

### Activity of MP16 in Dark-Induced Leaf Senescence

**FIGURE 4:**
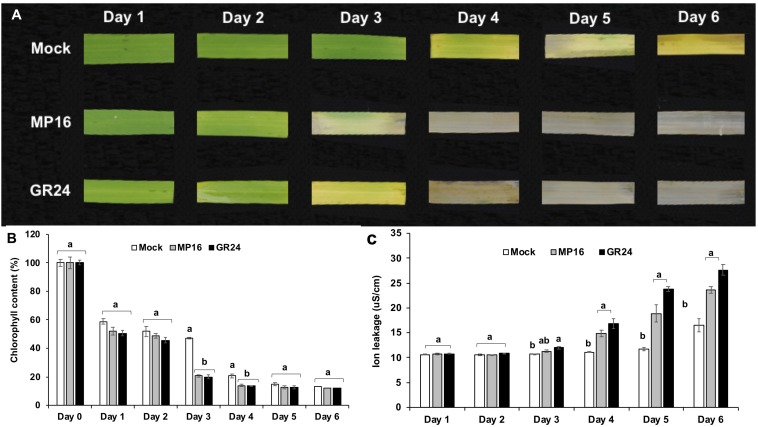
Dark-induced leaf senescence in response to MP16 and GR24. **(A)** Changes in rice leaf color for a period of 6 days. **(B)** Daily effect on chlorophyll content **(C)** Daily effect on membrane ion leakage. Data are means ± SE (*n* = 6). Means not sharing a letter in common differ significantly at *P*_0.05_.

The high activity of MP16 led us to investigate its activity in triggering dark-induced leaf senescence in comparison to the standard SL analog GR24. MP16 and GR24 treated leaf segments showed a loss of the green color on the third day after application, which was 1 day earlier than mock control (Figure 4A). Accordingly, measurement of the chlorophyll content unraveled a significant reduction in GR24 and MP16 treated segments on the third and fourth day of treatment (Figure 4B). In both GR24 and MP16 treated leaf segments, the ion leakage, an indicator of the loss of membrane integrity, began to increase on the third day of treatment and continued to increase till day 6 (Figure 4C).

### Parasitic Seed Germination in Response to MPs Application

**FIGURE 5:**
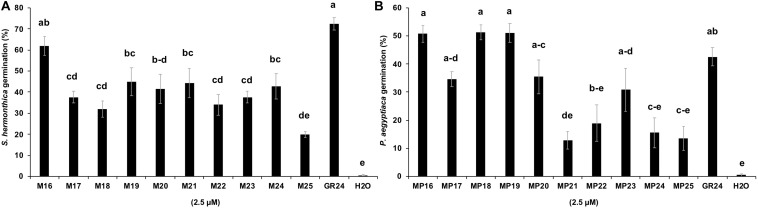
*Striga hermonthica* and *Phelipanche aegyptiaca* seed germination in response to MPs application. Each MP (2.5 μM) was applied in 55 μl volume on a disk containing 50–100 preconditioned **(A)** *S. hermonthica* **(B)** *P. aegyptiaca* seeds. GR24 and H_2_O are included as positive and negative control, respectively. Bars represent means ± SE (*n* = 6). Means not sharing a letter in common differ significantly at *P*_0.05_.

**FIGURE 6:**
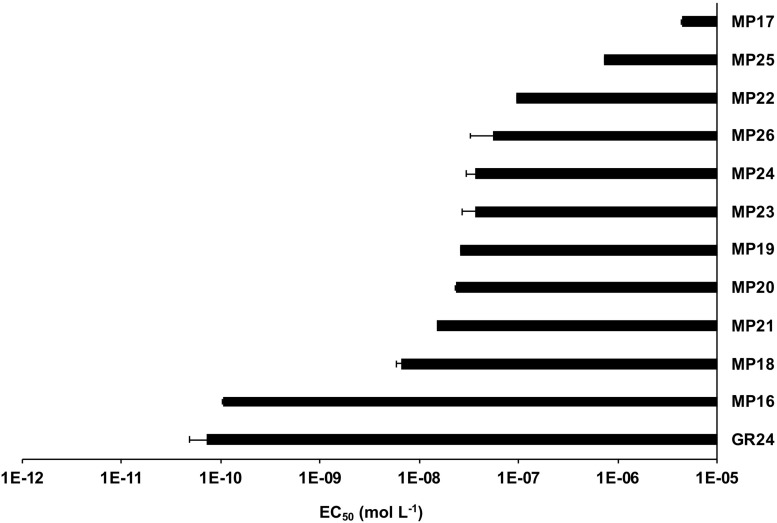
Half Maximal Effective Concentration (EC_50_) of MP16-MP25 for *Striga* seed germination. Preconditioned *S. hermonthica* seeds were treated with 55 μl of aqueous solutions of each MP, with various concentrations ranging from 10^–5^ to 10^–12^ M on a disk containing 50–100 pre-conditioned *Striga* seeds. GR24 is included as positive control.

Next, we tested the activity of the new SL analogs in inducing seed germination in root parasitic plants. For this purpose, we applied the compounds to pre-conditioned *S. hermonthica* seeds and determined the rate of germination (Figure 5A). Application of MP16 at a 2.5 μM concentration resulted in a high *Striga* germination rate (62%), which was comparable to that of GR24 (72%). Application of other MPs led generally to lower germination rate. We also tested germination inducing activity of all MPs on seeds of *Phelipanche aegyptiaca*. Application of MP16, MP18, and MP19 at 2.5 μM concentration caused highest germination rates (around 51%), which was statistically equal to that observed upon treatment with GR24 (43%) (Figure 5B). MP21, MP22, MP24, and MP25, showed the lowest activity in inducing *Phelipanche* seed germination. For a better quantification of *Striga* seed germination inducing activity, we determined the half maximal effective concentration (EC_50_) of the different SL analogs (Figure 6). The standard analog GR24 showed the lowest EC_50_ value of 7.23 × 10^–11^ mol L^–1^ for *Striga* germination. MP16 was the second-best analog in this assay, with an EC_50_ value of 1.05 × 10^–10^ mol L^–1^, followed by MP18 (EC_50_ value of 6.44 × 10^–9^ mol L^–1^). MP17 was the less active analog with the highest EC_50_ value of 4.35 × 10^–6^ mol L^–1^.

### Stability of SL Analogs

**FIGURE 7:**
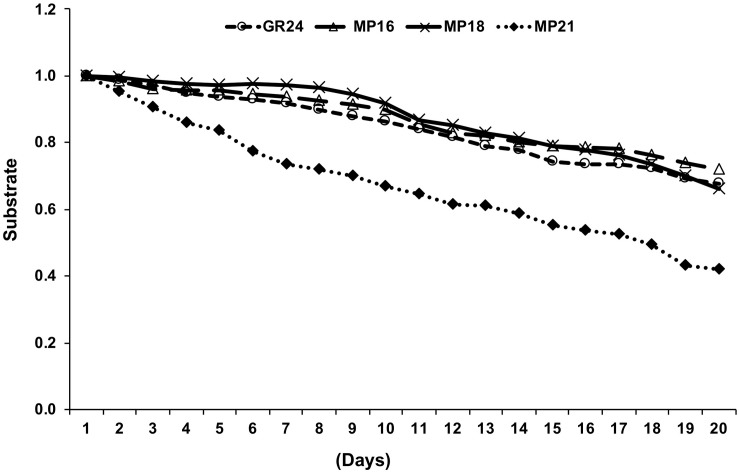
Measuring stability of selected MPs by UPLC. The relative amount of non-degraded MP16, MP18, MP21 and GR24 was monitored in HPLC on daily basis up to 3 weeks and determined by comparison with internal standard. Data are means ± SE (*n* = 3). X-axis [time (days)]; Y-axis (substrate).

Stability is an important feature that impacts biological activity of SL analogs. Hence, we determined the stability of MP16, MP18, the most active new MPs in *Striga* germination assays, and MP21, a less active MP, in aqueous solution (pH 5.5–6.0), in comparison with the standard SL analog GR24. The stability of MP16 and MP18 was in the range of that of GR24, while MP21 showed a higher degradation rate, compared to GR24 (Figure 7), which might be a reason for its low performance.

### ShHTL7/OsD14-Mediated YLG Hydrolysis Assays

**FIGURE 8:**
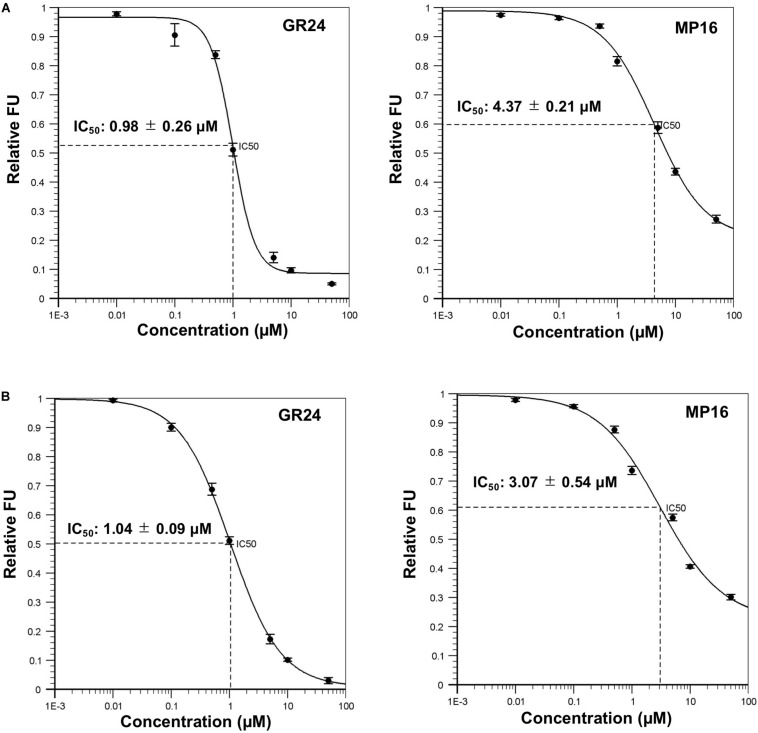
*In vitro* YLG (Yoshimulactone Green) hydrolysis assays. **(A)** YLG hydrolysis of GR24 and MP16 by ShHTL7. **(B)** YLG hydrolysis of GR24 and MP16 by OsD14. Seven concentrations of MP16 and GR24 ranging from 0.01, 0.1, 0.5, 1.0, 10, and 50 μM were applied, and YLG fluorescent intensity was measured (with or without purified ShHTL7 or OsD14). Values represent means ± SE (*n* = 3).

The high activity of MP16 in different bioassays led us to measure the rate of its hydrolysis by ShHTL7 (*Striga hermonthica* Hyposensitive to Light) (Toh et al., 2015), the most sensitive SL receptor in *Striga* seeds, and the rice SL receptor OsD14 (Arite et al., 2009; Yao et al., 2016). The hydrolysis rate of MP16 (IC_50_: 4.37 ± 0.21 μM) by ShHTL7 was lower than that of GR24 (IC_50_: 0.98 ± 0.26 μM) (Figure 8A). Similarly, in competition with YLG in OsD14-mediated hydrolysis assays, GR24 was hydrolyzed more efficiently (IC_50_: 1.04 ± 0.09 μM) than MP16 (IC_50_: 3.07 ± 0.54 μM) (Figure 8B).

## Discussion

SLs are an important plant hormone with an array of diverse functions including regulation of plant growth and development, adaptation to nutritional availability, contribution to biotic and abiotic stress responses, and communication with beneficial microorganisms and root parasitic plants (Cook et al., 1966; Gomez-Roldan et al., 2008; Ha et al., 2014; Al-Babili and Bouwmeester, 2015; Decker et al., 2017; Waters et al., 2017). However, the limited availability of SLs is a constraint in investigating their biology and a major obstacle on the way toward their application at a large scale in agriculture. The development of easy-to-synthesize, low cost SL analogs/mimics with improved bioactivity is a key in solving problem (Prandi and McErlean, 2019). Designing of SL analogs that exert particular SL functions would be also a decisive step in translating fundamental SL research into application (Zwanenburg and Pospisil, 2013). Up-till now GR24, which requires a complex synthesis protocol (Mangnus et al., 1992a, b), is the widely used SL analog in labs. The demand for simple SL analogs has led to the development of compounds, such as AR36 (Boyer et al., 2014), 4-Br debranone (4BD) (Fukui et al., 2011, 2013) and Nijmegen-1 (Wigchert et al., 1999), which, however, are generally less active than GR24. Similarly, SL mimics were developed that are lacking the ABC scaffold but contain only D-ring with an appropriate substitute at C-5 (Zwanenburg et al., 2013; Boyer et al., 2014; Prandi and McErlean, 2019). SL mimics, such as Debranones (Fukui et al., 2011) and Para-bromo- phenyloxy butanolide (Vurro et al., 2019) T010 (Samejima et al., 2016), showed weak to moderate bioactivity. However, these SL mimics provide a clue to develop simple SL with easy synthesis (Zwanenburg et al., 2016b). The discovery of SL biosynthesis intermediate carlactone (Alder et al., 2012) paved the way for the discovery of some non-canonical SLs like methyl carlactonoate (Abe et al., 2014). The presence of non-canonical SLs as a separate family of SLs was further supported by the identification of heliolactone (Ueno et al., 2014), zealactone, zeapyranolactone (Charnikhova et al., 2017, 2018), and avenaol (Yasui et al., 2017). Recently, we have developed MPs as analogs of non-canonical SLs, which are characterized by a simple structure and showed reasonable activity in different bioassays (Jamil et al., 2018). These encouraging results prompted us to develop a new series of MPs, with the aim of generating high-active and simple SL analogs. In this paper, we describe the synthesis and biological tests of these new MPs.

First, we evaluated the activity of these analogs (MP16–MP25) in regulating the growth and architecture of rice. Since shoot branching/tillering inhibition is the best known hormonal function of SLs (Gomez-Roldan et al., 2008; Umehara et al., 2008), we tested the effect of our MPs on this trait. For this purpose, we used the high tillering, SL deficient *d10*/CCD8 rice mutant. Six of the developed MPs (MP16 to MP21) were as efficient as GR24 in reducing the tiller number of this mutant (Figure 3). In addition to restoring wild-type tillering phenotype in *d10* mutant, application of MP16 retarded the growth, lowered plant height and accelerated leaf senescence of treated seedlings, leading to noticeable decrease in dry biomass, which was more pronounced than that observed with other SL analogs including GR24 (Supplementary Figure S2). The high activity of MP16 makes it a very good candidate for application as structurally simple growth regulator and points to its being a very efficient SL analog. In addition, it effect on plant height in *d10* background (Figure 3A), which contradicts that of other MPs and GR24, indicates that it might interfer with other SL-independent developmental processes. The retarded growth and senescence effect caused by MP16 further inspired us to test the effect of this analog on dark-induced leaf senescence activity (Yamada et al., 2014). Here again, we observed an activity similar to that of GR24, i.e., the application of MP16 accelerated the leaf senescence process, leading to a color loss after 2 days. Other senescence parameters, such as chlorophyll content and electrolytes leakage, were changed to a similar extent upon MP16 and GR24 application (Figures 4A–C).

Root parasitic plants, in particular *S. hermonthica*, are causing huge damage in African agriculture and are considered as a major threat to global food security. Infested fields have accumulated huge amounts of long-living and tiny seeds (Rubiales et al., 2009), which represent a major constraint in combating *Striga* and related species. Induction of suicidal germination of root parasitic seeds is a promising approach to combat these weeds in Africa and other parts of the world (Samejima et al., 2016; Zwanenburg et al., 2016a; Kountche et al., 2019). This approach requires efficient and easy-to-synthesize SL analogs and is a major potential application field for these chemistries. The results of *Striga* bioassays showed that application of 2.5 μM of MP16 caused 65% germination, which is in the range of the standard SL analog GR24 (Figure 5A). Among the previously described MPs (Jamil et al., 2018) MP1 was the most potent SL analog (EC_50_ = 1.5 × 10^–9^ mol L^–1^). This EC_50_ value was about 17 times higher than that of GR24. In the present study, we show that EC_50_ value of MP16 (EC_50_ = 1.05 × 10^–10^ mol L^–1^) is less than two times higher than that of GR24 (EC_50_ = 7.23 × 10^–11^ mol L^–1^). However, it can be speculated that the simple synthesis route of MP16 will more than compensate the lower activity of this compound, in comparison to GR24. In the near future, we are going to evaluate the efficiency of MP16 in heavily *Striga*-infested fields in Burkina Faso and Niger.

We also tested the utility of the new MPs in inducing seed germination of a further root parasitic weed, i.e., *Phelipanche aegyptiaca*. Here, we observed very high activity with MP16, MP18, and MP19, which was statistically equal to that of GR24 (Figure 5B). In contrast to *P. aegyptiaca*, we observed significant activity in inducing *S. hermonthica* seed germination only with MP16 (Figure 5A). This indicates that MPs vary in their efficiency in inducing seed germination of different parasitic weed species. The species-dependent distinction is likely caused by structural diversity of receptors involved in binding and perception of SLs or by differences in the uptake of particular MPs. Taken together, this result is a further indication for the application potential of the new MPs in combating different root parasitic species.

It is assumed that the hydrolysis rate of SLs by the *Striga* SL receptor ShHTL7 is an indicator for their activity in inducing seed germination. Therefore, we measured the hydrolysis of MP16 by conducting the competitive, ShHT7-mediated YLG hydrolysis assay. MP16 exhibited higher IC_50_ value (IC_50_ = 4.37 ± 0.21 μM) than GR24 (IC_50_ = 0.98 ± 0.26 μM), which is in line with its lower activity. Similarly, despite the fact that MP16 showed a growth inhibitory and senescence effect on rice seedlings, the OsD14-mediated YLG hydrolysis assays showed it as a less preferred substrate (IC_50_ = 3.07 ± 0.54 μM), compared to GR24 (IC_50_ = 1.04 ± 0.09 μM). This outcome reveals that some other elements, such as stability, transport and SL uptake could also be involved in the growth retarding activity of MP16 (Figure 8).

## Conclusion

In conclusion, we have developed a new series of SL analogs that will help in understanding the different functions of SLs and have a large application potential in agriculture, particularly in combating root parasitic weeds.

## Data Availability Statement

All datasets generated for this study are included in the article/Supplementary Material.

## Author Contributions

SA-B and MJ conceived and designed the experiments. IT, TO, and TA designed and synthesized the analogs. BK, JW, IH, and K-PJ assisted and performed the experiments. MJ, TA, SA-B, and others wrote the manuscript and respective parts. SA-B supervised the study. All authors read, edited, and approved the manuscript.

## Conflict of Interest

The authors declare that the research was conducted in the absence of any commercial or financial relationships that could be construed as a potential conflict of interest.

## Footnotes

1 http://www.ic50.tk/index.html

## Supplementary Material

FIGURE S1 Tillering inhibition of SL insensitive *d3* rice mutant by MP16-MP25. **(A)** Tillering phenotype of *d3* mutant in response to MPs. SL analogs were applied (2.5 μM) to 1 week old *d3* rice seedlings grown hydroponically in 50 ml tube twice a week up to 3 weeks. **(B)** Number of tillers per plant counted after 3 weeks of MPs application. **(C)** Dry biomass of *d3* rice seedlings measured after 3 weeks of MPs application. Data are means ± SE (*n* = 8). Means not sharing a letter in common differ significantly at *P*_0.05_.

FIGURE S2 Effect of MPs on *d10* rice plant height. All the MPs were applied (2.5 μM) to 1-week old hydroponically grown rice seedlings (*d10*/CCD8 mutant) twice a week up to 3 weeks. Plant height per plant were measured (*n* = 8) and compared by one-way ANOVA. Means not sharing a letter in common differ significantly at *P*_0.05_.
