# Supplementary figures and images for: A New Series of Carlactonoic Acid Based Strigolactone Analogs for Fundamental and Applied Research
Source: Front Plant Sci. 2020 Apr 15;11:434. doi: 10.3389/fpls.2020.00434 (PMC7179673; doi:10.3389/fpls.2020.00434)

## Slide 1
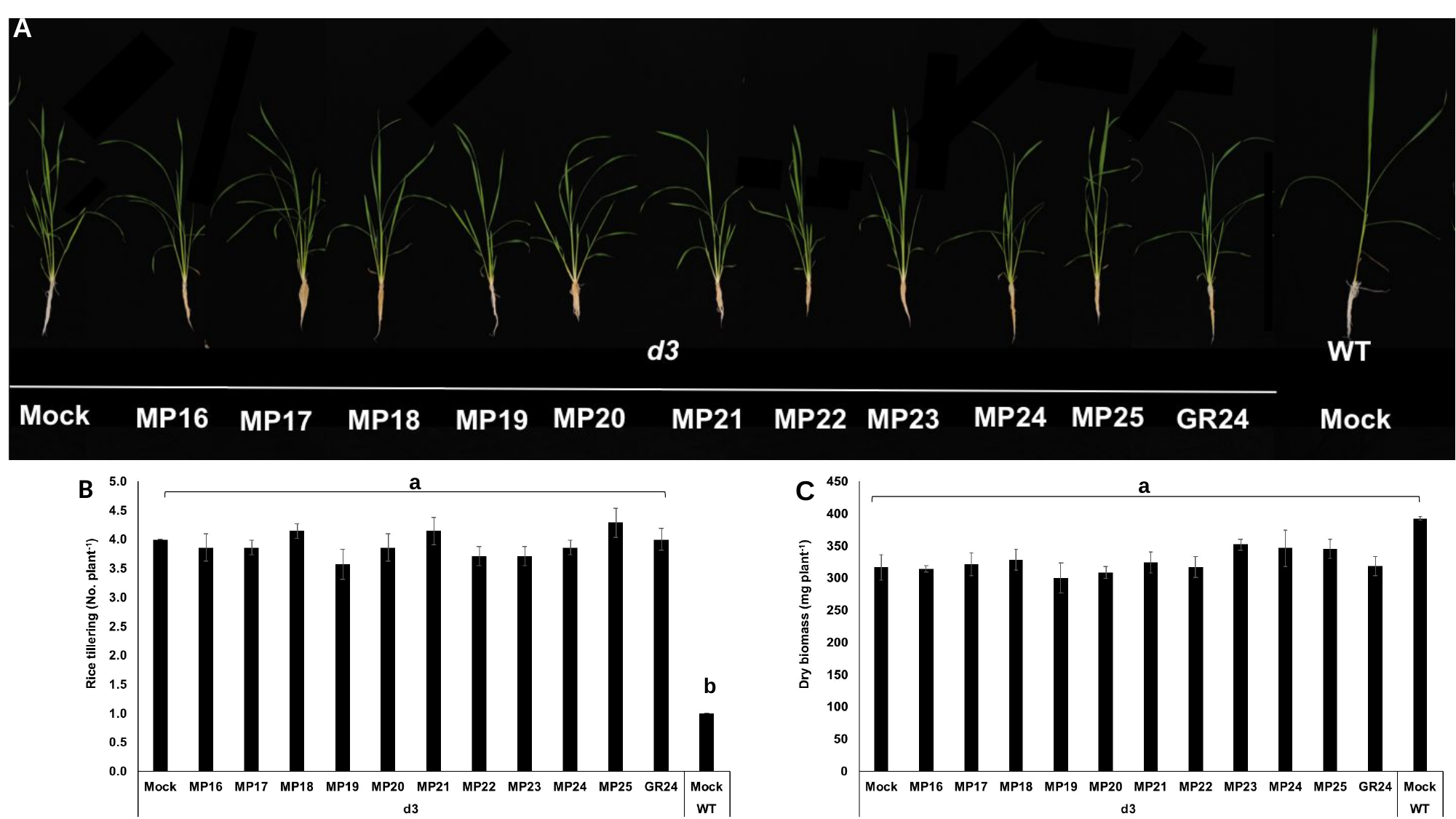

A
a
B
a
C
b

## Slide 2
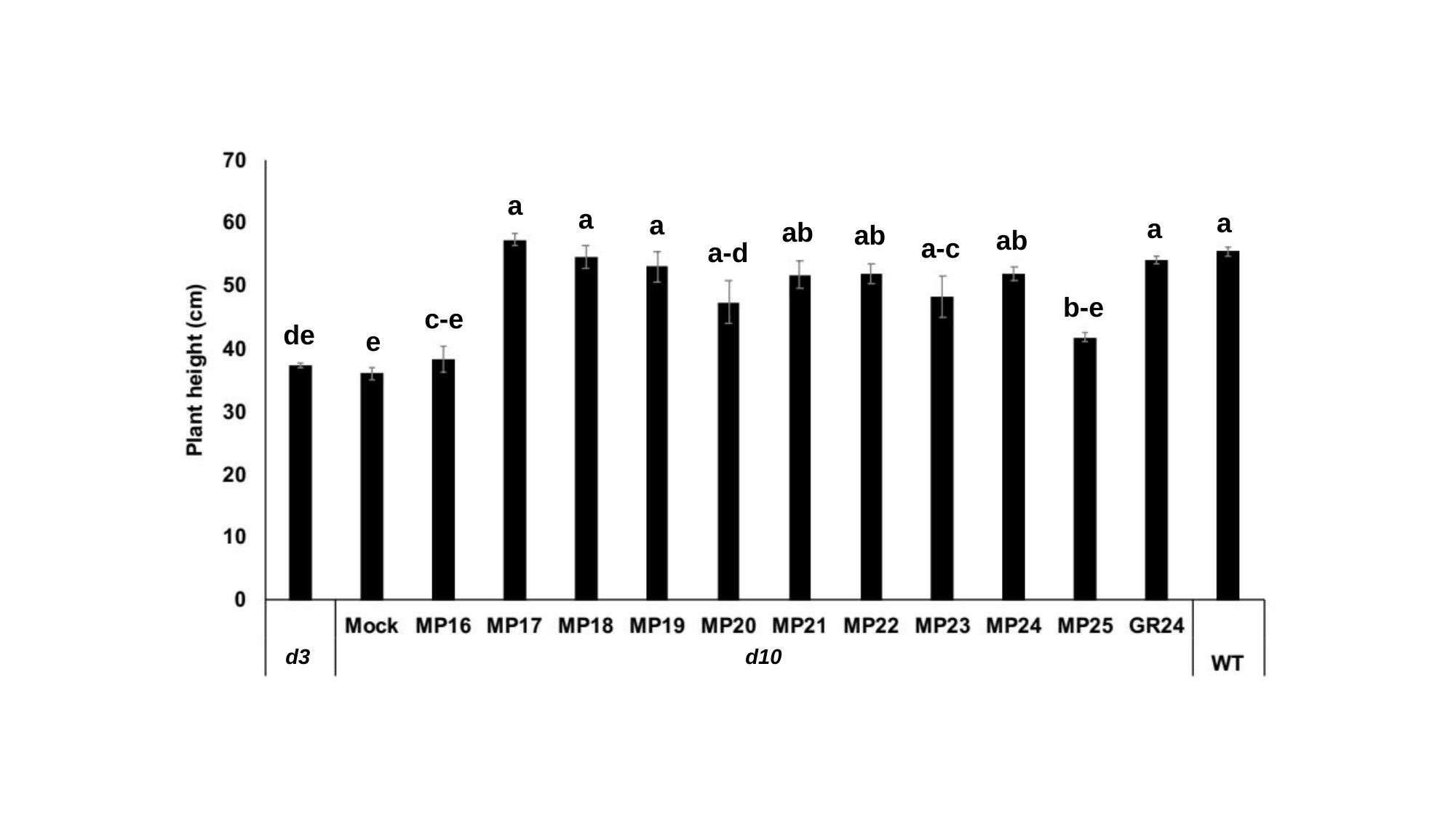

a
a
a
a
a
ab
ab
ab
a-c
a-d
b-e
c-e
de
e
d3
d10

Supplement: FIGURE S1 — Tillering inhibition of SL insensitive d3 rice mutant by MP16-MP25. (A) Tillering phenotype of d3 mutant in response to MPs. SL analogs were applied (2.5 μM) to 1 week old d3 rice seedlings grown hydroponically in 50 ml tube twice a week up to 3 weeks. (B) Number of tillers per plant counted after 3 weeks of MPs application. (C) Dry biomass of d3 rice seedlings measured after 3 weeks of MPs application. Data are means ± SE (n = 8). Means not sharing a letter in common differ significantly at P0.05. [file Presentation_1.PPTX]
